# Enhanced Adsorption and Recovery of Uranyl Ions by NikR Mutant-Displaying Yeast

**DOI:** 10.3390/biom4020390

**Published:** 2014-04-11

**Authors:** Kouichi Kuroda, Kazuki Ebisutani, Katsuya Iida, Takashi Nishitani, Mitsuyoshi Ueda

**Affiliations:** Division of Applied Life Sciences, Graduate School of Agriculture, Kyoto University, Sakyo-ku, Kyoto 606-8502, Japan; E-Mails: k_kuro@kais.kyoto-u.ac.jp (K.K.); ebisutani-kazuki@glico.co.jp (K.E.); k.iida5919@gmail.com (K.I.); takashi.nishitani@nagase.co.jp (T.N.)

**Keywords:** cell surface engineering, arming yeast, bioadsorption, uranyl ions, NikR

## Abstract

Uranium is one of the most important metal resources, and the technology for the recovery of uranyl ions (UO_2_^2+^) from aqueous solutions is required to ensure a semi-permanent supply of uranium. The NikR protein is a Ni^2+^-dependent transcriptional repressor of the nickel-ion uptake system in *Escherichia coli*, but its mutant protein (NikRm) is able to selectively bind uranyl ions in the interface of the two monomers. In this study, NikRm protein with ability to adsorb uranyl ions was displayed on the cell surface of *Saccharomyces cerevisiae*. To perform the binding of metal ions in the interface of the two monomers, two metal-binding domains (MBDs) of NikRm were tandemly fused via linker peptides and displayed on the yeast cell surface by fusion with the cell wall-anchoring domain of yeast α-agglutinin. The NikRm-MBD-displaying yeast cells with particular linker lengths showed the enhanced adsorption of uranyl ions in comparison to the control strain. By treating cells with citrate buffer (pH 4.3), the uranyl ions adsorbed on the cell surface were recovered. Our results indicate that the adsorption system by yeast cells displaying tandemly fused MBDs of NikRm is effective for simple and concentrated recovery of uranyl ions, as well as adsorption of uranyl ions.

## 1. Introduction

Uranium, an element from the actinide series, is one of the heaviest elements naturally occurring on earth, and is one of the most important metal resources used as nuclear fuel. The amount of minable uranium in the earth’s crust is estimated to be 5.5 million tons. Compared to other minor metals, uranium deposits are relatively widespread, with major world producers including Australia (23%), Kazakhstan (15%), Russia (10%), and others. Based on annual use, however, uranium is predicted to be depleted within 100 years. It is therefore important to develop novel uranium sources to maintain a stable energy supply. Seawater contains a considerable amount of uranium, and the amount estimated to be present in the oceans (4.6 billion tons) is comparable to 20 thousand years’ worth of usage. Therefore, the development of a uranium-recovering system from seawater could provide a virtually permanent supply of energy. However, it is difficult to recover uranium from seawater using existing techniques due to the low concentration of uranyl ions (3.3 parts per billion, ppb) in seawater.

For the recovery of metal ions from a solution containing metal ions at low concentrations, the bioadsorption of metal ions using microorganisms is an attractive technology. Notably, cell-surface adsorption by yeast cell surface engineering, which enables the display of functional proteins by fusion with cell wall-anchoring proteins, is an advantageous strategy [1,2,3,4,5]. The benefits of this technique include the capability to use mild treatment for adsorption/recovery, the easy recovery of metal ions from cells without cell breakage, and the reusability of the yeasts used in the adsorption/recovery. An additional advantage is that the cell-surface display system allows high-throughput screening of protein/peptide libraries owing to the direct evaluation of the displayed protein/peptide without purification and concentration.

In contrast to the d-block transition metals with crucial biological functions, actinides including uranium have limited biological activity despite their chemical reactivity. Uranium can be present in a number of oxidation states, of which the +6 oxidation state, such as uranyl ion (UO_2_^2+^) and its complexes, is the most stable under aerobic and aqueous conditions. Serum proteins including transferrin [6,7] have been identified to interact with uranyl ions; however, only a few reported cases include a detailed analysis. The protein capable of selective binding of uranyl ions was designed by site-directed mutagenesis of the *E. coli* Ni^2+^-responsive protein NikR [8]. NikR protein is a Ni^2+^-dependent transcriptional repressor of the nickel-ion uptake system in *E. coli* [9,10,11,12]. The tetrameric structure of NikR consists of a core of four metal-binding domains (MBDs) and two flanking ribbon-helix-helix DNA-binding domains (DBDs) [13]. NikR mutant (NikRm), that was reported to bind uranyl ions, has the three mutations in Val72, His76, and Cys95 [8]. To achieve the favorable coordination environment for uranyl ions, His76 and Cys95 were mutated to aspartic acid (H76D and C95D), leading to the coordination either in a monodentate or a bidentate fashion. In addition, for the accommodation of uranyl oxo groups, Val72 was mutated to serine (V72S), which has the potential to form a hydrogen bond with one of the oxo groups of the uranyl cation. Taking advantage of this information on enzyme engineering, NikRm was displayed on the yeast cell surface as a uranyl ion chelator by yeast cell surface engineering in this study. In the construction of surface-engineered yeasts for adsorption of metal ions, the adsorption ability of engineered cells depends on the properties of the displayed metal-binding proteins. Based on the crystal structure of NikR, nickel ions are bound in the interface of the two monomers [13]. Therefore, to allow for binding of metal ions in the interface, two MBDs of NikRm were tandemly fused via linker peptides and were displayed on the yeast cell surface by fusion with the cell wall-anchoring domain of yeast α-agglutinin.

In this study, we attempted to construct a cell-surface-engineered yeast with uranyl ions-binding capabilities as a recovery system for uranium from aqueous solutions such as seawater. The adsorption and recovery of uranyl ions by the yeasts displaying tandemly fused MBDs of NikRm were evaluated, and the applicability of cell-surface adsorption to the collection of uranyl ions is discussed.

## 2. Results and Discussion

### 2.1. Cell Surface Display of Tandemly Fused MBDs of NikR Mutant

**Figure 1:**
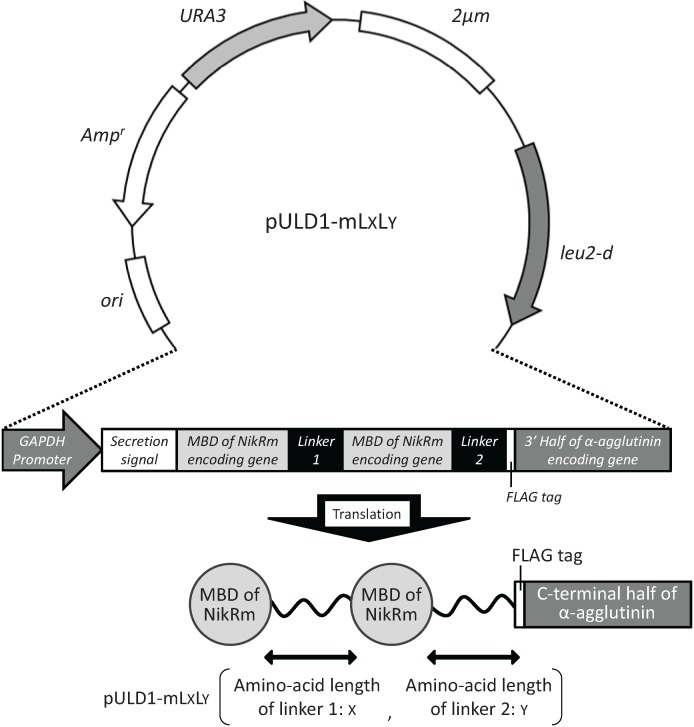
Plasmid pULD1-mLxLy constructed for displaying tandemly fused metal-binding domains(MBDs) of NikR mutant (NikRm) on the yeast cell surface.

Plasmids (pULD1-mLxLy) were constructed for cell surface display of tandemly fused MBDs of NikR mutant (NikRm) by fusion with α-agglutinin and FLAG tag for validating the display, as described in “Experimental” section. The constructed multi-copy plasmids contain the fusion gene composed of the glyceraldehydes-3-phosphate dehydrogenase (GAPDH) promoter, secretion signal of glucoamylase from *Rhizopus oryzae*, MBD of NikRm, linker 1 (see “Experimental” section), MBD of NikRm, linker 2 (see “Experimental” section), FLAG-tag, and *C*-terminal half of α-agglutinin as the cell-wall anchoring domain in this order (Figure 1). To optimize the coordination structure of tandemly fused MBDs of NikRm, pULD1-mLxLy was designed to possess different linker lengths of linkers 1 and 2. As a negative control, the multi-copy plasmid pULD1 displaying only the FLAG-tag was used in the bioadsorption experiment.

**Figure 2:**
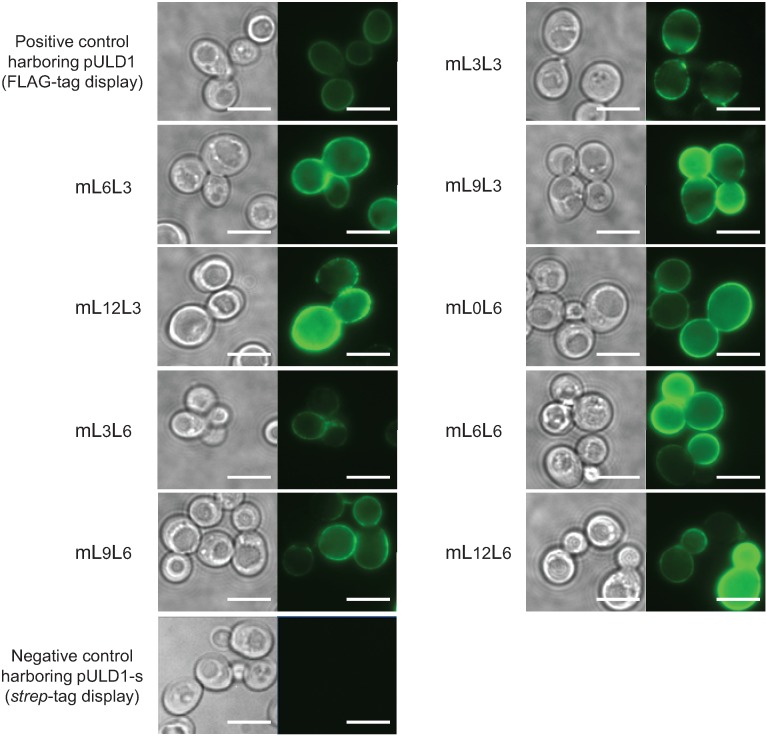
Immunofluorescent labeling of tandemly fused MBDs of NikRm displayed on the yeast cell surface using anti-FLAG antibody and Alexa Fluor 488 goat anti-mouse IgG antibody. Phase-contrast micrographs (**left**) and immunofluorescence micrographs (**right**). Yeast mLxLy is the constructed strain harboring pULD1-mLxLy, where x and y represent the amino-acid lengths of linkers 1 and 2, respectively. The scale bar indicates 5 μm.

To confirm the cell surface display of the tandemly fused MBDs of NikRm, immunofluorescent labeling of the transformants was performed (Figure 2). Yeast cells harboring the constructed plasmids showed green fluorescence derived from fluorescent antibodies binding on the cell surface, and negative control cells harboring the pULD1-s plasmid for the display of only a *strep*-tag showed no fluorescence. These results indicate that tandemly fused MBDs of NikRm with different linker lengths were successfully displayed on the yeast cell surface.

### 2.2. Bioadsorption of Uranyl Ions by Cell-Surface-Engineered Yeasts

**Figure 3:**
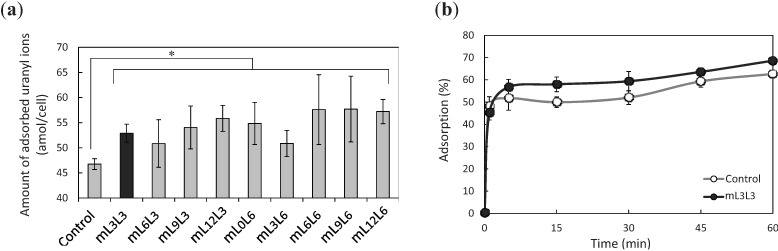
Cell-surface adsorption of uranyl ions by surface-engineered yeasts. (**a**) Uranyl ion adsorption on a single cell at pH 4.0 was quantified by dividing the number of adsorbed uranyl ions by the number of yeast cells; (**b**) Time-dependent changes in adsorption efficiency of uranyl ions at pH 4.0. Data represent the means ± S.E.M. of three independent experiments. * *p* < 0.05, determined by Dunnett’s test.

To evaluate the adsorption ability of the constructed yeast strains and to estimate the optimum combination of linker lengths, the adsorption of uranyl ions in a mixed solution containing 100 ppb of Be, Bi, Co, In, and U was assessed using the yeast cells displaying tandemly fused MBDs of NikRm (Figure 3a). A comparison of adsorption abilities showed significant differences between the control strain harboring pULD1 and all other strains displaying tandemly fused MBDs of NikRm. Among the constructed strains, the strain mL3L3, for which the adsorption ability data had the smallest standard deviation, was used in experiments to further evaluate the enhanced adsorption of uranyl ions. To investigate the optimum conditions of uranyl ion adsorption, the adsorption efficiencies at various adsorption times were measured (Figure 3b). The time required to reach adsorption equilibrium was shorter in the experiment (Figure 3b) than in the screening experiment (Figure 3a). The adsorption of uranyl ions started just after the input of yeast cells into metal solution and was completed within 5 min, suggesting that the adsorption after just one minute was sufficient.

The optimum combination of linker lengths between two MBDs and between MBD and α-agglutinin for the effective and stable adsorption of uranyl ions was determined. Among the constructed combinations, mL3L3, which has three amino acids of each linker, showed the most stable adsorption of uranyl ions. Despite being one of the shorter linker length combinations, mL3L3, may have been sufficient to accommodate the coordination sphere because the structural flexibility offered by the tandemly fused MBDs of NikRm was improved by removing the *N*-terminal domain of NikRm.

The minimum time required for adsorption was examined by measuring the adsorption efficiencies at various adsorption times. As shown in Figure 3b, the adsorption reaction on the cell surface proceeded rapidly, and the time required to show significant differences in adsorption abilities of the mL3L3 yeast *vs.* the control strain was 15 min. The rapid reaction observed may be due to a high-accessibility of uranyl ions to the displayed MBDs in comparison with the intracellular accumulation of ions via the innate metal uptake system of living organism. This property is advantageous to a uranium-recovery system from aqueous solutions such as seawater as an industrial application, as it leads to short operating times.

**Figure 4:**
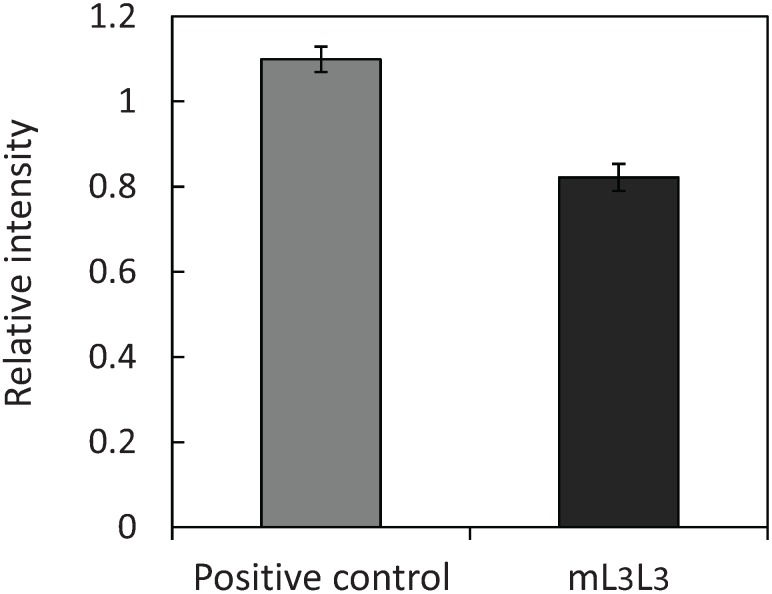
Immunofluorescence intensities of control and mL3L3 yeast strains. The fluorescence intensities of the cells after immunofluorescent labeling were measured. Data represent the means ± S.E.M. of three independent experiments.

To investigate whether the amount of adsorbed uranyl ions attributed to the surface-displayed MBDs of NikRm, the number of tandemly fused MBDs of NikRm on the cell surface was evaluated by measuring fluorescence intensity after immunofluorescent labeling (Figure 4) carried out as described in “Experimental” section. Fluorescence intensity was determined by subtracting the fluorescence intensity of the negative control strain harboring the pULD1-s plasmid from the intensities obtained for each engineered yeast strain. Our results show that the tandemly fused MBDs of NikRm were displayed effectively on the yeast cell surface with an efficiency comparable to the positive control strain of FLAG-tag display, and the number of the tandemly fused MBDs of NikRm displayed on the mL3L3 yeast was estimated to be 7.5 × 10^5^ molecules per single cell. Because tandemly fused MBDs can bind two uranyl ions, the number of uranyl ions potentially adsorbed per cell is estimated as 1.5 × 10^6^ molecules. When calculated from the result shown in Figure 3a, the number of adsorbed uranyl ions in mL3L3 cells was higher than in control cells by 2.9 × 10^6^ molecules per cell. The experimentally determined number of adsorbed uranyl ions was thus twice the estimated number. Moreover, the actual number of the displayed proteins is thought to be greater than estimated by immunofluorescent labeling and the synergistic effect between the displayed protein and cell wall components could be generated.

### 2.3. Recovery of Uranyl Ions Adsorbed on Yeast Cell Surface

**Figure 5:**
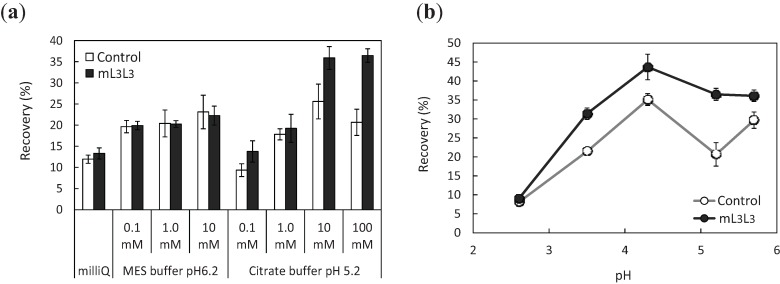
Recovery efficiencies of uranyl ions adsorbed on control and mL3L3 yeast strains. (**a**) Recovery of the adsorbed uranyl ions by ultrapure water (milliQ), MES buffer (pH 6.2), and citrate buffer (pH 5.2) at various concentrations (0.1–100 mM); (**b**) pH-dependence of recovery efficiencies using 100 mM citrate buffer (indicated pHs). Data represent the means ± S.E.M. of three independent experiments.

To recover uranyl ions adsorbed on the cell surface of engineered yeast, cells were treated with various concentrations of either ultrapure water, 2-morpholinoethanesulfonic acid (MES; pH 6.2), or citrate buffer (pH 5.2) subsequent to adsorption (Figure 5a). Uranyl ions released from the cells were quantified by inductively coupled plasma mass spectrometry (ICP-MS), and the recovery efficiency (the ratio of the amount of released uranyl ions to that of initial uranyl ions) was measured. The results indicate that the higher concentrations of release solutions led to increased recovery efficiency, and citrate buffer was more effective than MES buffer as a release solution. The explanation for this difference is that the three carboxy groups of citrate acted as chelating agents. For further enhancement of recovery efficiency, the effect of the pH of the citrate buffer on recovery efficiency was investigated (Figure 5b). The number of uranyl ions recovered from cell surface by treating with citrate buffer (pH 4.3) was 44% of the number of uranyl ions in the initial solution (pH 4.3). At low pH, the recovery of uranyl ions was low because of a decrease in the complex formation constant, which is characteristic of low pH ranges. The optimum pH of the citrate buffer for high recovery (pH 4.3) was effective in the release of uranyl ions from the yeast cell surface: The ratio of the number of recovered uranyl ions to the number of adsorbed uranyl ions was 77%, as calculated from an adsorption ratio of 57% of initial uranyl ions, and a recovery ratio of 44% of initial uranyl ions. Considering the difficulty of improving and optimizing release conditions, further enhancement of recovery efficiency would have to be achieved by further improvements of the adsorption efficiency.

Cell surface adsorption of metal ions by cell surface engineering is a promising strategy for the recovery of uranyl ions from aqueous solutions. In this study, uranyl ions were not only adsorbed but also recovered easily from the cell surface by a mild treatment without cell disruption, unlike in conventional intracellular accumulation methods. Therefore, surface-engineered yeasts could be repeatedly used as bioadsorbents of uranyl ions. Such a recyclable recovery system would be highly advantageous to the sustainability of natural resources such as uranyl ions in modern societies.

## 3. Experimental

### 3.1. Strains and Media

*E. coli* DH5α [F*^−^*, *ϕ*80d*lacZ∆*M15, *∆*(*lac*ZYA-*arg*F)U169, *hsd*R17(*r_K_*^−^, *m_K_^+^*), *rec*A1, *end*A1, *deo*R, *thi*-1, *sup*E44, *gyr*A96, *rel*A1, *λ^−^*] was used as a host cell for recombinant DNA manipulation. *Saccharomyces cerevisiae* BY4741/*Δsed1* (*MAT***a**, *his3Δ1*, *leu2Δ0*, *met15Δ0*, *ura3Δ0*, *YDR077w::KanMX4*; Euroscarf, Frankfurt, Germany) was used as the host cell for the efficient display of proteins on the cell surface [14]. *E. coli* was grown in Luria-Bertani medium (1% (*w*/*v*) tryptone, 0.5% yeast extract, 0.5% sodium chloride) containing 100 μg/mL ampicillin. Yeast cells were precultivated aerobically in synthetic dextrose medium (SDC + HML) (0.67% (*w*/*v*) yeast nitrogen base without amino acids, 2% glucose, 0.5% casamino acids, 0.002% l-histidine, 0.002% l-methionine, and 0.003% l-leucine) buffered by 100 mM 2-morpholinoethanesulfonic acid (MES) at pH 6.8, and then cultivated in the same medium.

### 3.2. Construction of Plasmids for Cell Surface Display

All primers used in the plasmid construction are listed in Table 1. NikR is a tetrameric protein consisting of two domains: DNA-binding domain (DBD) and metal-binding domain (MBD). The DNA fragment encoding the *C*-terminal 85 amino acids of NikR including MBD was amplified from *E. coli* DH5α genomic DNA by PCR using primers NikR-MBD-F and NikR-MBD-R. The amplified DNA fragment was digested with *Bgl* II and *Xho* I, and then inserted into the *Bgl* II-*Xho* I section of the pULD1 plasmid [14] for the efficient display of proteins on the yeast cell surface. The resulting plasmid was named pULD1-NikR-MBD. Triple simultaneous substitutions of amino acids in the MBD (V72S, H76D, and C95D) [8] of NikR were conducted by site-directed mutagenesis for selective coordination of uranyl ions, and the resulting NikR mutant was named NikRm. The plasmid pULD1-NikR-MBD was used as a template for site-directed mutagenesis using six primers: V72S-F, V72S-R, H76D-F, H76D-R, C95D-F, and C95D-R. The introduction of these mutations was confirmed by the dideoxynucleotide chain termination method. To bind metal ions in the interface of two MBDs, two MBDs of NikRm were tandemly fused via linker peptides consisting of various numbers of amino acids by *Bgl* II and *Sal* I cohesive termini (Figure 1). The DNA fragments encoding the MBD of NikRm with three or six amino acid linkers at the *C*-terminus were amplified by PCR using the primers *Bgl* II-*Sal* I-NikRm-forward and NikRm-L3-R, or NikRm-L6-R, respectively. The amplified DNA fragments were digested with *Bgl* II and *Xho* I, and inserted in the *Bgl* II-*Xho* I section of the pULD1 plasmid. The constructed plasmids were named pULD1-mL3 and pULD1-mL6, respectively. The DNA fragments encoding MBDs of NikRm with linkers of different lengths (0, 3, 6, 9, or 12 amino acids) were amplified by PCR using the forward primer *Bgl* II-*Sal* I-NikRm-F and reverse primers NikRm-L0-R, NikRm-L3-R, NikRm-L6-R, NikRm-L9-R, or NikRm-L12-R, respectively. Amplified DNA fragments were digested with *Bgl* II and *Xho* I, and inserted into the *Bgl* II-*Sal* I section of pULD1-mL3 or pULD1-mL6 (*Xho* I and *Sal* I have compatible cohesive ends). The constructed plasmids for the cell surface display of the tandemly fused MBDs of NikRm were named pULD1-mLxLy (x: Amino-acid length of linker 1 between two MBDs of NikRm, y: Amino-acid length of linker 2 between MBD of NikRm and anchoring domain of α-agglutinin). The plasmid pULD1-s [14] for the cell surface display of the *strep*-tag and pULD1 for the cell surface display of the FLAG-tag were used as negative controls of immunofluorescence labeling and bioadsorption experiments, respectively.

**Table 1:** Primers used in this study.

Primer name	Sequence	Application
NikR-MBD-F	5'-CGATAGATCTGGCACGCAAGGTTTCGCGGTGCTGTC-3'	Cloning of NikR-MBD
NikR-MBD-R	5'-CGATCTCGAGATCTTCCTTCGGCAAGCACTGC-3'	Cloning of NikR-MBD
V72S-F	5'-CGCGACTTAGCCAGCCGCATTAGCTCCACCCAGGATCATCACCACGACC-3'	Amino acid substitution (V72S) of NikR-MBD
V72S-R	5'-GGTCGTGGTGATGATCCTGGGTGGAGCTAATGCGGCTGGCTAAGTCGCG-3'	Amino acid substitution (V72S) of NikR-MBD
H76D-F	5'-GCATTGTCTCTCCACCCAGGATCATCACCACGACCTCTCCG-3'	Amino acid substitution (H76D) of NikR-MBD
H76D-R	5'-CGGAGAGGTCGTGGTGATGATCCTGGGTGGAGACAATCA-3'	Amino acid substitution (H76D) of NikR-MBD
C95D-F	5'-GCACATCAACCACGACGACGACCTGGAAATCGCCGTG-3'	Amino acid substitution (C95D) of NikR-MBD
C95D-R	5'-CACGGCGATTTCCAGGTCGTCGTCGTGGTTGATGTGC-3'	Amino acid substitution (C95D) of NikR-MBD
*Bgl* II-*Sal* I-NikRm-F	5'-CGATAGATCTAGAGTCGACGGCACGCAAGGTTTCGCGGTGCTGTCG-3'	Cloning of MBD-NikRm with linker
NikRm-L0-R	5'-CGATCTCGAGATCTTCCTTCGGCAAGCACTGC-3'	Cloning of MBD-NikRm with linker
NikRm-L3-R	5'-CGATCTCGAGAGATCCACCATCTTCCTTCGGCAAGCACTGC-3'	Cloning of MBD-NikRm with linker
NikRm-L6-R	5'-CGATCTCGAGAGATCCACCAGATCCACCATCTTCCTTCGGCAAGCACTGC-3'	Cloning of MBD-NikRm with linker
NikRm-L9-R	5'-CGATCTCGAGAGATCCACCAGATCCACCAGATCCACCATCTTCCTTCGGCAAGCACTGC-3'	Cloning of MBD-NikRm with linker
NikRm-L12-R	5'-CGATCTCGAGAGATCCACCAGATCCACCAGATCCACCAGATCCACCATCTTCCTTCGGCAAGCACTGC-3'	Cloning of MBD-NikRm with linker

### 3.3. Transformation of S. cerevisiae

The lithium acetate method [15] was used for the transformation of *S. cerevisiae* cells. Transformants were isolated by incubation on a plate of SDC + HML medium at 30 °C for 48 h. All constructed yeast strains are listed in Table 2.

**Table 2:** Yeast strains constructed in this study.

Strain name	Signature	Length of linker 1 (a.a.)	Length of linker 2 (a.a.)
mL3L3	Displaying tandemly fused MBDs of NikRm	3	3
mL6L3	Displaying tandemly fused MBDs of NikRm	6	3
mL9L3	Displaying tandemly fused MBDs of NikRm	9	3
mL12L3	Displaying tandemly fused MBDs of NikRm	12	3
mL0L6	Displaying tandemly fused MBDs of NikRm	0	6
mL3L6	Displaying tandemly fused MBDs of NikRm	3	6
mL6L6	Displaying tandemly fused MBDs of NikRm	6	6
mL9L6	Displaying tandemly fused MBDs of NikRm	9	6
mL12L6	Displaying tandemly fused MBDs of NikRm	12	6
pULD1	Negative control for bioadsorption experiment	−	−
pULD1-s	Negative control for immunofluorescence labeling	−	−

### 3.4. Immunofluorescence Microscopy

To confirm the display of tandemly fused MBDs of NikRm on the yeast cell surface, immunofluorescent labeling of cells was performed using the FLAG epitope tag as described previously [5]. Cells were incubated in phosphate-buffered saline (PBS; pH 7.4) containing 1% bovine serum albumin for 30 min prior to immunostaining. Mouse monoclonal anti-FLAG M2 antibody (Sigma, St. Louis, MO, USA) was used as the primary antibody at a dilution of 1:300. A mixture of cells and the antibody was incubated using a rotator for 1.5 h at room temperature. Cells were then washed with PBS (pH 7.4). Alexa Fluor 488-conjugated goat anti-mouse IgG antibody (Invitrogen, Carlsbad, CA, USA) diluted at 1:300 was then incubated with the cells using a rotator for 1.5 h at room temperature. After washing with PBS (pH 7.4), cells were suspended in 30 μL of PBS (pH 7.4) and observed by inverted microscope IX71 (Olympus, Tokyo, Japan) through a U-MNIBA2 mirror unit with a BP470-490 excitation filter, PM505 dichroic mirror, and BA510-550 emission filter (Olympus). Live images were obtained using Aqua-Cosmos 2.0 software (Hamamatsu Photonics, Shizuoka, Japan) controlling a digital charge-coupled device camera (C4742-95-12ER, Hamamatsu Photonics).

### 3.5. Measurement of Fluorescence Intensity

To estimate the number of tandemly fused MBDs of NikRm that were displayed on the yeast cell surface, the fluorescence intensity of cells was evaluated following immunofluorescent labeling. The immunofluorescence-labeled cells were precipitated by centrifugation at 800× *g*, and cell densities were adjusted to an optical density of 3.0 at 600 nm (OD_600_) with PBS (pH 7.4). Fluorescence intensity was measured using a Fluoroskan Ascent FL fluorometer (Labsystems, Helsinki, Finland) in a tissue-culture plate (353072 Multi-well 96-well; Becton-Dickinson, Franklin Lakes, NJ, USA) using excitation and emission wavelengths of 485 nm and 527 nm respectively. The measured values of fluorescence intensity correspond to the number of the displayed protein on the cell surface.

### 3.6. Bioadsorption and Recovery of Uranyl Ions by Cell-Surface-Engineered Yeast Cells

The bioadsorption of metal ions by the cell surface-engineered yeasts was measured according to the previously describe protocol [5]. Prior to adsorption, the cell-surface-engineered yeasts were precultivated and cultivated in SDC + HML medium (pH 6.8) buffered by 100 mM MES at 30 °C for 24 h. The cells were collected by centrifugation at 800× *g* for 10 min. The cells were incubated in a metal solution containing 100 ppb of Be, Bi, Co, In, and U that was prepared by diluting 1 mg/L custom assurance standard (SPEX CertiPrep, XSTC-289, Metuchen, NJ, USA) with ultrapure water to an OD_600_ of 0.25. The adsorption reaction was performed at room temperature with rotary shaking and centrifuged at 800× *g* for 10 min. The number of residual uranyl ions in the supernatant was quantified by ICP-MS (Agilent 7500cx, Agilent Technologies, Santa Clara, CA, USA). For adsorption, the pH of the metal solution was adjusted to 4.0 with HNO_3_ or NaOH. The resulting cell pellets were treated with ultrapure water, MES buffer, or citrate buffer as release solutions to recover cell-surface-bound uranyl ions. A mixture of cells and release solution was incubated at room temperature with rotary shaking followed by centrifugation at 800× *g* for 10 min. The concentration of uranyl ions released into the resulting supernatant was also measured by ICP-MS. The adsorption efficiency and the recovery efficiency were calculated as follows (Ci, initial metal concentration; Cs, metal concentration of supernatant after bioadsorption experiment; Cr, metal concentration after recovery experiment):

Adsorption efficiency (%) = (Ci − Cs)/Ci × 100(1)

Recovery efficiency (%) = Cr/Ci × 100(2)

## 4. Conclusions

The results of our current study suggest that cell-surface-engineered yeast cells displaying tandemly fused MBDs of NikRm are effective in the adsorption of uranyl ions, which are the oxyanion form of uranium present in seawater. Our adsorption technique is important to the future of stable energy supply, since uranium is used as nuclear fuel. The development of cell-surface adsorption systems by yeast cell surface engineering would be advantageous for the adsorption and recovery of various metal ions, and may contribute to the effective recycling of valuable metal ions from metal-containing water in industrial applications as well as in natural environments.
